# Poly[[[diisothio­cyanato­cobalt(II)]-bis­[μ-4-*tert*-butyl-2,6-bis­(1,2,4-triazol-1-ylmeth­yl)phenol]] dimethyl­formamide disolvate dihydrate]

**DOI:** 10.1107/S1600536809005121

**Published:** 2009-02-18

**Authors:** Zhao-lian Chu

**Affiliations:** a Institute of Molecular Engineering & Applied Chemistry, School of Chemistry and Chemical Engineering, Anhui University of Technology, Maanshan 243002, People’s Republic of China

## Abstract

In the title compound, {[Co(NCS)_2_(C_16_H_20_N_6_O)_2_]·2C_3_H_7_NO·2H_2_O}_*n*_, each Co^II^ ion located on an inversion center is six-coordinated by four equatorial N atoms from four different 4-*tert*-butyl-2,6-bis­(1,2,4-triazol-1-ylmeth­yl)phenol (*L*) ligands, and by two N atoms from two axial thio­cyanate anions [Co—N = 2.104 (3)–2.144 (3) Å]. The metal centres are connected *via* the bidentate *L* ligands into two-dimensional polymeric layers parallel to *bc* plane. The dimethyl­formamide and solvent water mol­ecules participate in inter­molecular O—H⋯O and O—H⋯S hydrogen bonds, which consolidate the crystal packing.

## Related literature

For related structures, see: Chu *et al.* (2007 ▶, 2008 ▶); Ma *et al.* (2003 ▶); Zhu *et al.* (2004 ▶, 2007 ▶). For details of the synthesis, see Yan *et al.* (1994 ▶). 
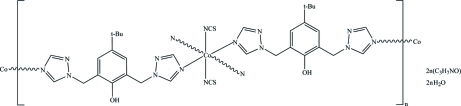


## Experimental

### 

#### Crystal data

[Co(NCS)_2_(C_16_H_20_N_6_O)_2_]·2C_3_H_7_NO·2H_2_O
*M* *_r_* = 982.07
Monoclinic, 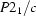

*a* = 12.561 (4) Å
*b* = 20.660 (6) Å
*c* = 10.571 (3) Å
β = 112.992 (5)°
*V* = 2525.2 (12) Å^3^
*Z* = 2
Mo *K*α radiation
μ = 0.48 mm^−1^
*T* = 291 K
0.30 × 0.30 × 0.20 mm

#### Data collection

Bruker SMART CCD area-detector diffractometer
Absorption correction: multi-scan (*SADABS*; Bruker, 2000 ▶) *T*_min_ = 0.869, *T*_max_ = 0.910
13505 measured reflections
4950 independent reflections
2902 reflections with *I* > 2σ(*I*)
*R*_int_ = 0.057

#### Refinement

*R*[*F* ^2^ > 2σ(*F* ^2^)] = 0.055
*wR*(*F* ^2^) = 0.123
*S* = 0.90
4950 reflections
301 parameters
H-atom parameters constrained
Δρ_max_ = 0.45 e Å^−3^
Δρ_min_ = −0.28 e Å^−3^

### 

Data collection: *SMART* (Bruker, 2000 ▶); cell refinement: *SAINT* (Bruker, 2000 ▶); data reduction: *SAINT*; program(s) used to solve structure: *SHELXS97* (Sheldrick, 2008 ▶); program(s) used to refine structure: *SHELXL97* (Sheldrick, 2008 ▶); molecular graphics: *SHELXTL* (Sheldrick, 2008 ▶) and *DIAMOND* (Brandenburg, 1998 ▶); software used to prepare material for publication: *SHELXTL*.

## Supplementary Material

Crystal structure: contains datablocks I, global. DOI: 10.1107/S1600536809005121/cv2519sup1.cif

Structure factors: contains datablocks I. DOI: 10.1107/S1600536809005121/cv2519Isup2.hkl

Additional supplementary materials:  crystallographic information; 3D view; checkCIF report

## Figures and Tables

**Table 1:** Hydrogen-bond geometry (Å, °)

*D*—H⋯*A*	*D*—H	H⋯*A*	*D*⋯*A*	*D*—H⋯*A*
O1—H1⋯O2	0.82	1.94	2.689 (4)	152
O2—H2*A*⋯O3	0.85	1.81	2.655 (5)	179
O2—H2*B*⋯S1^i^	0.85	2.51	3.321 (3)	161

Symmetry code: (i) 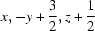.

**Fig. 1.**
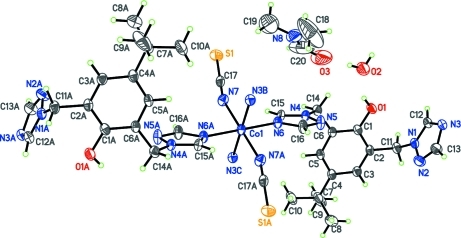
A portion of the crystal structure of the title compound, showing 30% probability displacement ellipsoids and the atom-numbering [symmetry codes: (A) -x + 1, -y + 1, -z + 1; (B) -x + 1, y - 1/2, -z + 3/2; (C) x, -y + 3/2, z - 1/2].

## supplementary crystallographic information

### Comment

Ligand 2,6-bis(1,2,4-triazol-1-ylmethyl)-4-*tert*-butyl-phenol (bttp) has been used to generate various metal-organic architectures with different transitional metal ions due to its polydentate character and bridging ability (Chu *et al.*, 2007, 2008; Ma *et al.*, 2003; Zhu *et al.*, 2004, 2007). As a further study of such complexes, the title Co^II^ complex is reported in this paper.

Each Co^II^ atom exhibits a slightly distorted octahedral environment with four nitrogen atoms from the triazole groups of four bttp ligands in the equatorial plane, and two nitrogen atoms from two thiocyanate ligands at the axial positions (Fig. 1). Each ligand adopts a *cis* conformation in which two triazole groups are on the same direction of the central phenyl ring. The dihedral angles between the phenyl ring and the two triazole rings are 97.8 (3) ° and 88.8 (3) °, respectively. The two triazole rings are inclined to one another, with a dihedral angle of 65.3 (3) °. Each bttp serves as a bidentate bridging ligand *via* two exodentate nitrogen atoms at the 4-position of the triazole rings while the nitrogen atoms at 1,2-positions remain uncoordinated. In this way four metal atoms and four bttp ligands form a 48-membered [*M*_4_*L*_4_] metallocyclic ring, which is further assembled into a two-dimensional network *via* Co–N coordination bonds (Fig. 2). The Co···Co distance linked by the bridged bttp ligand is 11.604 (1) Å. The water oxygen atom is uncoordinated, and contributes to the formation of O–H···O and O–H···S hydrogen-bonding interactions with phenol group and DMF molecule (Table 1).

### Experimental

All solvents and chemicals were of analytical grade and were used without further purification. Ligand bttp was prepared *via* a one-step Mannich reaction as a white powder in 57% yield (Yan *et al.*, 1994). For the synthesis of title compoud, a solution of bttp (0.1 mmol), Co(NO_3_)_2_.6H_2_O (0.1 mmol) and NH_4_SCN (0.25 mmol) in 30 ml e thanol was refluxed for 2 h, and then cooled to room temperature and filtered. The collected solid was dissolved in 1 ml DMF, and 20 ml e thanol was added to this solution. The mixture was left to stand at room temperature for two weeks and pink crystalline products were obtained (30.5 mg, 62%). Anal. Calcd. for C_40_H_58_CoN_16_O_6_S_2_: C, 48.92; H, 5.95; N, 22.82. Found: C, 48.88; H, 5.98; N, 22.72.

### Refinement

All H atoms were  geometrically positioned (C–H 0.93–0.97 Å, O–H 0.82–0.85 Å), and refined as riding, with U_iso_(H)=1.2-1.5 U_eq_  of the parent atom.

### Crystal data

**Table:**

[Co(NCS)_2_(C_16_H_20_N_6_O)_2_]·2C_3_H_7_NO·2H_2_O	*F*(000) = 1034
*M**_r_* = 982.07	*D*_x_ = 1.292 Mg m^−^^3^
Monoclinic, *P*2_1_/*c*	Mo *K*α radiation, λ = 0.71073 Å
Hall symbol: -P 2ybc	Cell parameters from 2950 reflections
*a* = 12.561 (4) Å	θ = 2.2–26.3°
*b* = 20.660 (6) Å	µ = 0.48 mm^−^^1^
*c* = 10.571 (3) Å	*T* = 291 K
β = 112.992 (5)°	Block, pink
*V* = 2525.2 (12) Å^3^	0.30 × 0.30 × 0.20 mm
*Z* = 2	

### Data collection

**Table:**

Bruker SMART CCD area-detector diffractometer	4950 independent reflections
Radiation source: fine-focus sealed tube	2902 reflections with *I* > 2σ(*I*)
graphite	*R*_int_ = 0.057
φ and ω scans	θ_max_ = 26.0°, θ_min_ = 2.0°
Absorption correction: multi-scan (*SADABS*; Bruker, 2000)	*h* = −15→9
*T*_min_ = 0.869, *T*_max_ = 0.910	*k* = −22→25
13505 measured reflections	*l* = −12→13

### Refinement

**Table:**

Refinement on *F*^2^	Primary atom site location: structure-invariant direct methods
Least-squares matrix: full	Secondary atom site location: difference Fourier map
*R*[*F*^2^ > 2σ(*F*^2^)] = 0.055	Hydrogen site location: inferred from neighbouring sites
*wR*(*F*^2^) = 0.123	H-atom parameters constrained
*S* = 0.90	*w* = 1/[σ^2^(*F*_o_^2^) + (0.0486*P*)^2^] where *P* = (*F*_o_^2^ + 2*F*_c_^2^)/3
4950 reflections	(Δ/σ)_max_ = 0.001
301 parameters	Δρ_max_ = 0.45 e Å^−^^3^
0 restraints	Δρ_min_ = −0.28 e Å^−^^3^

### Special details

**Table:**

Experimental. The structure was solved by direct methods (Bruker, 2000) and successive difference Fourier syntheses.
Geometry. All e.s.d.'s (except the e.s.d. in the dihedral angle between two l.s. planes) are estimated using the full covariance matrix. The cell e.s.d.'s are taken into account individually in the estimation of e.s.d.'s in distances, angles and torsion angles; correlations between e.s.d.'s in cell parameters are only used when they are defined by crystal symmetry. An approximate (isotropic) treatment of cell e.s.d.'s is used for estimating e.s.d.'s involving l.s. planes.
Refinement. Refinement of *F*^2^ against ALL reflections. The weighted *R*-factor *wR* and goodness of fit *S* are based on *F*^2^, conventional *R*-factors *R* are based on *F*, with *F* set to zero for negative *F*^2^. The threshold expression of *F*^2^ > σ(*F*^2^) is used only for calculating *R*-factors(gt) *etc*. and is not relevant to the choice of reflections for refinement. *R*-factors based on *F*^2^ are statistically about twice as large as those based on *F*, and *R*- factors based on ALL data will be even larger.

### Fractional atomic coordinates and isotropic or equivalent isotropic displacement parameters (Å^2^)

**Table:**

	*x*	*y*	*z*	*U*_iso_*/*U*_eq_	
Co1	0.5000	0.5000	0.5000	0.03695 (19)	
C1	0.5771 (3)	0.86800 (14)	0.5064 (3)	0.0393 (8)	
C2	0.6792 (3)	0.90337 (13)	0.5400 (3)	0.0404 (8)	
C3	0.7690 (3)	0.87839 (14)	0.5126 (3)	0.0433 (8)	
H3	0.8360	0.9029	0.5346	0.052*	
C4	0.7636 (3)	0.81724 (14)	0.4528 (3)	0.0420 (8)	
C5	0.6623 (3)	0.78350 (14)	0.4230 (3)	0.0404 (8)	
H5	0.6563	0.7426	0.3841	0.048*	
C6	0.5691 (3)	0.80680 (13)	0.4473 (3)	0.0371 (8)	
C7	0.8633 (3)	0.79381 (16)	0.4164 (4)	0.0550 (10)	
C8	0.9756 (3)	0.7951 (2)	0.5444 (5)	0.0955 (15)	
H8A	1.0379	0.7796	0.5216	0.143*	
H8B	0.9678	0.7678	0.6138	0.143*	
H8C	0.9916	0.8386	0.5783	0.143*	
C9	0.8743 (5)	0.8385 (2)	0.3075 (5)	0.1115 (19)	
H9A	0.8033	0.8381	0.2275	0.167*	
H9B	0.9363	0.8239	0.2832	0.167*	
H9C	0.8902	0.8818	0.3432	0.167*	
C10	0.8440 (4)	0.72489 (18)	0.3605 (5)	0.0915 (15)	
H10A	0.7729	0.7229	0.2806	0.137*	
H10B	0.8397	0.6960	0.4295	0.137*	
H10C	0.9070	0.7123	0.3360	0.137*	
C11	0.6889 (3)	0.97044 (14)	0.6015 (3)	0.0478 (9)	
H11A	0.7630	0.9889	0.6125	0.057*	
H11B	0.6290	0.9977	0.5379	0.057*	
C12	0.5946 (3)	0.99424 (14)	0.7654 (3)	0.0455 (8)	
H12	0.5296	1.0151	0.7034	0.055*	
C13	0.7154 (3)	0.95306 (17)	0.9391 (4)	0.0597 (10)	
H13	0.7526	0.9391	1.0294	0.072*	
C14	0.4596 (3)	0.76755 (13)	0.4002 (3)	0.0447 (9)	
H14A	0.4007	0.7913	0.4189	0.054*	
H14B	0.4318	0.7607	0.3018	0.054*	
C15	0.4614 (3)	0.64600 (13)	0.4173 (3)	0.0421 (8)	
H15	0.4319	0.6366	0.3239	0.050*	
C16	0.5281 (3)	0.63924 (14)	0.6298 (3)	0.0507 (9)	
H16	0.5557	0.6217	0.7178	0.061*	
C17	0.2724 (3)	0.51848 (14)	0.2245 (4)	0.0446 (9)	
C18	0.1450 (9)	0.6297 (5)	0.6463 (9)	0.298 (8)	
H18A	0.2127	0.6063	0.7034	0.448*	
H18B	0.0774	0.6090	0.6486	0.448*	
H18C	0.1493	0.6733	0.6795	0.448*	
C19	0.0711 (6)	0.5834 (3)	0.4258 (8)	0.178 (3)	
H19A	−0.0069	0.5866	0.4200	0.267*	
H19B	0.1015	0.5414	0.4597	0.267*	
H19C	0.0720	0.5897	0.3362	0.267*	
C20	0.1849 (6)	0.6730 (4)	0.4695 (12)	0.223 (6)	
H20	0.1732	0.6685	0.3775	0.267*	
N1	0.6785 (2)	0.97134 (11)	0.7339 (3)	0.0424 (7)	
N2	0.7593 (3)	0.94366 (14)	0.8470 (3)	0.0633 (9)	
N3	0.6134 (2)	0.98416 (11)	0.8952 (3)	0.0421 (7)	
N4	0.4785 (2)	0.70493 (10)	0.4699 (2)	0.0381 (7)	
N5	0.5213 (3)	0.70182 (11)	0.6081 (3)	0.0537 (8)	
N6	0.4925 (2)	0.60231 (11)	0.5167 (3)	0.0419 (7)	
N7	0.3582 (3)	0.50307 (12)	0.3096 (3)	0.0498 (7)	
N8	0.1387 (4)	0.6307 (2)	0.5147 (7)	0.1162 (19)	
O1	0.4893 (2)	0.89753 (10)	0.5304 (3)	0.0546 (6)	
H1	0.4390	0.8709	0.5237	0.082*	
O2	0.3105 (2)	0.83963 (13)	0.5642 (3)	0.1002 (11)	
H2A	0.2885	0.8004	0.5513	0.120*	
H2B	0.2559	0.8631	0.5668	0.150*	
O3	0.2404 (4)	0.7173 (2)	0.5249 (8)	0.239 (4)	
S1	0.14960 (9)	0.54051 (5)	0.10273 (12)	0.0765 (4)	

### Atomic displacement parameters (Å^2^)

**Table:**

	*U*^11^	*U*^22^	*U*^33^	*U*^12^	*U*^13^	*U*^23^
Co1	0.0484 (4)	0.0262 (3)	0.0367 (4)	−0.0040 (3)	0.0171 (3)	0.0003 (3)
C1	0.051 (2)	0.0312 (17)	0.0407 (19)	0.0049 (16)	0.0238 (17)	0.0051 (14)
C2	0.055 (2)	0.0296 (16)	0.043 (2)	−0.0029 (16)	0.0258 (18)	−0.0028 (14)
C3	0.049 (2)	0.0362 (17)	0.049 (2)	−0.0073 (16)	0.0249 (18)	−0.0029 (15)
C4	0.056 (2)	0.0307 (17)	0.044 (2)	0.0001 (16)	0.0247 (18)	0.0025 (14)
C5	0.055 (2)	0.0258 (16)	0.041 (2)	0.0040 (16)	0.0191 (17)	−0.0010 (14)
C6	0.049 (2)	0.0241 (15)	0.0377 (18)	0.0004 (15)	0.0166 (16)	0.0046 (13)
C7	0.064 (3)	0.042 (2)	0.073 (3)	0.0009 (18)	0.041 (2)	−0.0084 (18)
C8	0.066 (3)	0.093 (3)	0.134 (4)	0.004 (3)	0.046 (3)	−0.024 (3)
C9	0.165 (5)	0.082 (3)	0.156 (5)	0.028 (3)	0.137 (4)	0.023 (3)
C10	0.092 (3)	0.060 (3)	0.146 (4)	0.000 (2)	0.072 (3)	−0.033 (3)
C11	0.062 (2)	0.0335 (17)	0.060 (2)	−0.0091 (17)	0.037 (2)	−0.0046 (16)
C12	0.054 (2)	0.0373 (18)	0.048 (2)	0.0070 (17)	0.0233 (18)	−0.0025 (16)
C13	0.061 (3)	0.071 (3)	0.045 (2)	0.012 (2)	0.019 (2)	0.0028 (19)
C14	0.053 (2)	0.0302 (17)	0.047 (2)	0.0053 (15)	0.0148 (18)	0.0054 (14)
C15	0.054 (2)	0.0330 (17)	0.0365 (19)	−0.0066 (16)	0.0143 (17)	−0.0067 (14)
C16	0.081 (3)	0.0337 (18)	0.039 (2)	−0.0038 (18)	0.0247 (19)	0.0002 (15)
C17	0.057 (2)	0.0317 (18)	0.050 (2)	−0.0062 (16)	0.027 (2)	−0.0054 (15)
C18	0.396 (17)	0.376 (16)	0.121 (7)	0.258 (14)	0.099 (9)	0.043 (8)
C19	0.159 (7)	0.117 (5)	0.234 (9)	0.029 (5)	0.051 (7)	0.011 (6)
C20	0.093 (6)	0.113 (6)	0.475 (19)	0.010 (5)	0.125 (9)	0.039 (9)
N1	0.0509 (19)	0.0322 (14)	0.0500 (18)	−0.0032 (13)	0.0263 (16)	−0.0068 (13)
N2	0.058 (2)	0.075 (2)	0.059 (2)	0.0159 (17)	0.0251 (18)	−0.0012 (17)
N3	0.0483 (18)	0.0381 (15)	0.0432 (18)	0.0033 (13)	0.0215 (14)	−0.0014 (12)
N4	0.0478 (18)	0.0279 (13)	0.0381 (17)	−0.0048 (12)	0.0163 (14)	−0.0010 (11)
N5	0.089 (2)	0.0319 (15)	0.0385 (17)	−0.0037 (15)	0.0229 (16)	−0.0047 (12)
N6	0.0578 (18)	0.0286 (13)	0.0404 (16)	−0.0038 (13)	0.0202 (14)	0.0003 (12)
N7	0.056 (2)	0.0466 (16)	0.0410 (17)	−0.0040 (16)	0.0127 (15)	0.0024 (14)
N8	0.065 (3)	0.065 (3)	0.192 (6)	0.003 (2)	0.022 (3)	−0.008 (3)
O1	0.0581 (16)	0.0363 (12)	0.0824 (18)	−0.0020 (12)	0.0416 (15)	−0.0086 (12)
O2	0.087 (2)	0.082 (2)	0.157 (3)	−0.0222 (17)	0.075 (2)	−0.0334 (19)
O3	0.106 (4)	0.096 (3)	0.512 (10)	−0.033 (3)	0.118 (5)	−0.063 (5)
S1	0.0555 (7)	0.0812 (7)	0.0776 (8)	0.0190 (6)	0.0094 (6)	−0.0002 (6)

### Geometric parameters (Å, °)

**Table:**

Co1—N7^i^	2.104 (3)	C12—N3	1.314 (4)
Co1—N7	2.104 (3)	C12—H12	0.9300
Co1—N6	2.126 (2)	C13—N2	1.306 (4)
Co1—N6^i^	2.126 (2)	C13—N3	1.344 (4)
Co1—N3^ii^	2.144 (3)	C13—H13	0.9300
Co1—N3^iii^	2.144 (3)	C14—N4	1.461 (3)
C1—O1	1.368 (4)	C14—H14A	0.9700
C1—C2	1.396 (4)	C14—H14B	0.9700
C1—C6	1.397 (4)	C15—N4	1.321 (3)
C2—C3	1.370 (4)	C15—N6	1.323 (4)
C2—C11	1.515 (4)	C15—H15	0.9300
C3—C4	1.403 (4)	C16—N5	1.310 (3)
C3—H3	0.9300	C16—N6	1.339 (4)
C4—C5	1.375 (4)	C16—H16	0.9300
C4—C7	1.526 (5)	C17—N7	1.147 (4)
C5—C6	1.380 (4)	C17—S1	1.641 (4)
C5—H5	0.9300	C18—N8	1.361 (8)
C6—C14	1.504 (4)	C18—H18A	0.9600
C7—C9	1.523 (5)	C18—H18B	0.9600
C7—C10	1.525 (5)	C18—H18C	0.9600
C7—C8	1.526 (5)	C19—N8	1.391 (7)
C8—H8A	0.9600	C19—H19A	0.9600
C8—H8B	0.9600	C19—H19B	0.9600
C8—H8C	0.9600	C19—H19C	0.9600
C9—H9A	0.9600	C20—O3	1.161 (9)
C9—H9B	0.9600	C20—N8	1.243 (8)
C9—H9C	0.9600	C20—H20	0.9300
C10—H10A	0.9600	N1—N2	1.356 (4)
C10—H10B	0.9600	N3—Co1^iv^	2.144 (3)
C10—H10C	0.9600	N4—N5	1.347 (3)
C11—N1	1.456 (4)	O1—H1	0.8200
C11—H11A	0.9700	O2—H2A	0.8500
C11—H11B	0.9700	O2—H2B	0.8501
C12—N1	1.311 (4)		
			
N7^i^—Co1—N7	180.0	C2—C11—H11A	108.8
N7^i^—Co1—N6	89.94 (10)	N1—C11—H11B	108.8
N7—Co1—N6	90.06 (10)	C2—C11—H11B	108.8
N7^i^—Co1—N6^i^	90.06 (10)	H11A—C11—H11B	107.7
N7—Co1—N6^i^	89.94 (10)	N1—C12—N3	111.9 (3)
N6—Co1—N6^i^	180.000 (1)	N1—C12—H12	124.0
N7^i^—Co1—N3^ii^	89.21 (11)	N3—C12—H12	124.0
N7—Co1—N3^ii^	90.79 (11)	N2—C13—N3	115.9 (3)
N6—Co1—N3^ii^	92.79 (9)	N2—C13—H13	122.0
N6^i^—Co1—N3^ii^	87.21 (9)	N3—C13—H13	122.0
N7^i^—Co1—N3^iii^	90.79 (11)	N4—C14—C6	111.3 (2)
N7—Co1—N3^iii^	89.21 (11)	N4—C14—H14A	109.4
N6—Co1—N3^iii^	87.21 (9)	C6—C14—H14A	109.4
N6^i^—Co1—N3^iii^	92.79 (9)	N4—C14—H14B	109.4
N3^ii^—Co1—N3^iii^	180.0	C6—C14—H14B	109.4
O1—C1—C2	116.5 (3)	H14A—C14—H14B	108.0
O1—C1—C6	124.4 (3)	N4—C15—N6	110.2 (3)
C2—C1—C6	119.1 (3)	N4—C15—H15	124.9
C3—C2—C1	120.0 (3)	N6—C15—H15	124.9
C3—C2—C11	120.0 (3)	N5—C16—N6	115.4 (3)
C1—C2—C11	120.0 (3)	N5—C16—H16	122.3
C2—C3—C4	122.4 (3)	N6—C16—H16	122.3
C2—C3—H3	118.8	N7—C17—S1	180.0 (4)
C4—C3—H3	118.8	N8—C18—H18A	109.5
C5—C4—C3	116.0 (3)	N8—C18—H18B	109.5
C5—C4—C7	124.0 (3)	H18A—C18—H18B	109.5
C3—C4—C7	119.9 (3)	N8—C18—H18C	109.5
C4—C5—C6	123.8 (3)	H18A—C18—H18C	109.5
C4—C5—H5	118.1	H18B—C18—H18C	109.5
C6—C5—H5	118.1	N8—C19—H19A	109.5
C5—C6—C1	118.8 (3)	N8—C19—H19B	109.5
C5—C6—C14	118.9 (3)	H19A—C19—H19B	109.5
C1—C6—C14	122.2 (3)	N8—C19—H19C	109.5
C9—C7—C10	108.7 (3)	H19A—C19—H19C	109.5
C9—C7—C4	108.9 (3)	H19B—C19—H19C	109.5
C10—C7—C4	111.8 (3)	O3—C20—N8	129.7 (12)
C9—C7—C8	109.6 (4)	O3—C20—H20	115.1
C10—C7—C8	108.2 (3)	N8—C20—H20	115.1
C4—C7—C8	109.7 (3)	C12—N1—N2	109.1 (3)
C7—C8—H8A	109.5	C12—N1—C11	129.2 (3)
C7—C8—H8B	109.5	N2—N1—C11	121.7 (3)
H8A—C8—H8B	109.5	C13—N2—N1	101.9 (3)
C7—C8—H8C	109.5	C12—N3—C13	101.2 (3)
H8A—C8—H8C	109.5	C12—N3—Co1^iv^	129.1 (2)
H8B—C8—H8C	109.5	C13—N3—Co1^iv^	129.2 (2)
C7—C9—H9A	109.5	C15—N4—N5	110.1 (2)
C7—C9—H9B	109.5	C15—N4—C14	129.5 (3)
H9A—C9—H9B	109.5	N5—N4—C14	120.4 (2)
C7—C9—H9C	109.5	C16—N5—N4	102.0 (2)
H9A—C9—H9C	109.5	C15—N6—C16	102.3 (2)
H9B—C9—H9C	109.5	C15—N6—Co1	128.4 (2)
C7—C10—H10A	109.5	C16—N6—Co1	129.1 (2)
C7—C10—H10B	109.5	C17—N7—Co1	160.7 (3)
H10A—C10—H10B	109.5	C20—N8—C18	123.5 (8)
C7—C10—H10C	109.5	C20—N8—C19	119.1 (9)
H10A—C10—H10C	109.5	C18—N8—C19	117.2 (8)
H10B—C10—H10C	109.5	C1—O1—H1	109.5
N1—C11—C2	113.7 (3)	H2A—O2—H2B	109.5
N1—C11—H11A	108.8		

Symmetry codes: (i) −*x*+1, −*y*+1, −*z*+1; (ii) −*x*+1, *y*−1/2, −*z*+3/2; (iii) *x*, −*y*+3/2, *z*−1/2; (iv) −*x*+1, *y*+1/2, −*z*+3/2.

### Hydrogen-bond geometry (Å, °)

**Table:**

*D*—H···*A*	*D*—H	H···*A*	*D*···*A*	*D*—H···*A*
O1—H1···O2	0.82	1.94	2.689 (4)	152
O2—H2A···O3	0.85	1.81	2.655 (5)	179
O2—H2B···S1^v^	0.85	2.51	3.321 (3)	161

Symmetry codes: (v) *x*, −*y*+3/2, *z*+1/2.
